# Pine Resin as a Natural Polymer Binder in Pine Cone-Reinforced Lightweight Concrete

**DOI:** 10.3390/polym18030364

**Published:** 2026-01-29

**Authors:** Celal Kistak, Araz Muhammed Hassan, Ayse Bicer, Nevin Celik

**Affiliations:** 1 Department of Mechanical Engineering, Firat University, Elazig 23119, Turkey; ckistak@firat.edu.tr (C.K.); eng.araz@gmail.com (A.M.H.); 2 Department of Bioengineering, Turgut Ozal University, Malatya 44210, Turkey; ayse.bicer@ozal.edu.tr

**Keywords:** pine tree cone, pine tree resin, lightweight concrete, thermal-mechanical properties

## Abstract

The aim of this study is to investigate the potential applications of pine cones as plant-based waste material in the construction industry. In order to achieve this target, the pine cone particles (PCP) are mixed with cement to create new lightweight concretes. Furthermore, pine tree resin (PTR), acting as a natural bio-polymer binder, is incorporated into selected samples to ascertain its potential as a binder. The pine cones are cut into particles of 2–4 cm, 0–2 cm, and ground into a powder. A series of critical tests is conducted on the novel produced samples, including thermal conductivity, specific heat, density, compressive strength, water absorption rate, and drying rate. The experiments show that thermal conductivity, specific heat capacity, and thermal expansion coefficient decrease as the weight ratio and size of PCP increase. The presence of PTR increases porosity, further decreasing thermal conductivity, specific heat, and thermal expansion coefficients for the majority of samples. The compressive strength values decrease with the presence of PTR and PCP. Regarding durability, the water absorption ratios remain below the critical 30% threshold, making the material suitable for internal applications or external facades protected by coating/plaster or as external coverings.

## 1. Introduction

In recent decades, the construction industry has faced growing pressure to adopt sustainable and eco-friendly materials to address global environmental challenges. Traditional insulation and construction materials, such as polyurethane foam, extruded polystyrene, expanded polystyrene foam, glass wool, and rock wool, are widely used for their effectiveness. However, these materials rely on non-renewable resources, require energy-intensive production processes, and pose long-term environmental risks [1,2]. Increasing concerns over resource depletion, pollution, and high energy demands have shifted researchers’ focus toward alternative materials derived from renewable and recyclable sources.

One promising alternative is plant-based biomass waste, particularly pine cone particles (PCP)s and pine tree resin (PTR), which are abundant and renewable resources. The PCPs, a by-product of forest management activities, are generated in large quantities globally. Unfortunately, they are often discarded, burned, or left in the environment, where they contribute to fire risks, attract pests, and negatively impact soil health. Despite being low-cost, renewable resource-rich in cellulose, hemicellulose, and lignin, PCPs remain largely underutilized. This study aims to evaluate their potential as sustainable additives for producing lightweight concrete.

Existing research on the use of pine cones in composites and concrete is limited but offers valuable insights. For example, Bayraktar et al. [3] produced alkali-activated foam concrete by using fly ash and pine cone powder as partial replacements for granulated blast-furnace slag and silica sand, respectively. The effect of varying pine cone powder and flying ash amounts on density, porosity, water absorption, flowability, thermal conductivity, high-temperature resistance, sorptivity, compressive strength, flexural strength, and resistance to sulfate attack was tested. The results showed that the new mixture exhibited the highest mechanical performance, lowest sorptivity, highest dry unit weight, and best high-temperature resistance.

Singh et al. [4] investigated the mechanical and thermophysical properties of the pine cone as coarse aggregate in newly produced concretes. The compressive strength, splitting tensile and flexural strength, and modulus of elasticity of pine cone concrete were found to be 34.98%, 30.23%, 24.94%, and 33.78% lower than those of concrete without pine cones.

Arrakhiz et al. [5] used pine cone fibers to reinforce thermoplastic composites, demonstrating excellent mechanical and thermal properties. Agayev and Ozdemir [6] used powdered pine cones in the concrete to improve mechanical and thermal properties. Additional studies by Efe [7] and Basturk et al. [8] confirmed that pine cone addition enhances hardness in bio-composites and epoxy resin composites. Ayrilmis et al. [9] used pine cone powder in medium-density fiberboard and found that formaldehyde emissions and water resistance were improved.

Synthetic polymers such as latex and epoxy are commonly used to improve concrete properties. However, natural bio-polymers such as pine tree resin offer a sustainable alternative. In this context, pine tree resin functions as a natural bio-polymer admixture that can modify the cementitious matrix. Beyond pine cones, pine tree resin, which is a kind of bio-resin, has shown promise as a binder in concrete. Bicer and Kar [10] studied concretes containing apricot resin and expanded polystyrene aggregates, finding favorable thermal and mechanical properties. McSwiggan et al. [11] evaluated bio-based resin as a substitute for standard epoxy in reinforced concrete, reporting comparable bond strength and durability. Kaya and Kar [12] and Bicer [13] also concluded that bio-resins can reduce thermal conductivity while maintaining acceptable mechanical strength in concrete.

Despite these findings, the use of PCP and PTR in lightweight concrete remains underexplored. This study seeks to address this gap by investigating the use of pine cones as PCP and PTR in cementitious materials. Unlike previous studies that investigated these materials separately, this research analyzes the synergistic effect of classified PCPs (0–2 cm, 2–4 cm, and powder) combined with PTR solution in a single cementitious matrix. The PCP is added into the mixture of the concrete at weight ratios (wt) of 20%, 40%, 60%, and 80%, along with 1% PTR as a binder. The samples are tested for mechanical and thermal properties, including compressive strength, thermal conductivity, thermal expansion coefficient, specific heat capacity, density, and water absorption to assess their suitability for residential, industrial, and commercial construction applications.

By leveraging the renewable and recyclable nature of PCP and PTR, this study aims to create cost-effective, environmentally friendly alternatives to conventional lightweight concrete. The findings are expected to address waste management challenges while contributing to the development of lightweight construction materials for low-load applications. This research aligns with global efforts to reduce environmental impact and foster innovation in material science and construction practices [2,9,10,14].

## 2. Materials and Methods

### 2.1. Preparing the Pine Cone Particles (PCP) for Testing

**Figure 1:**
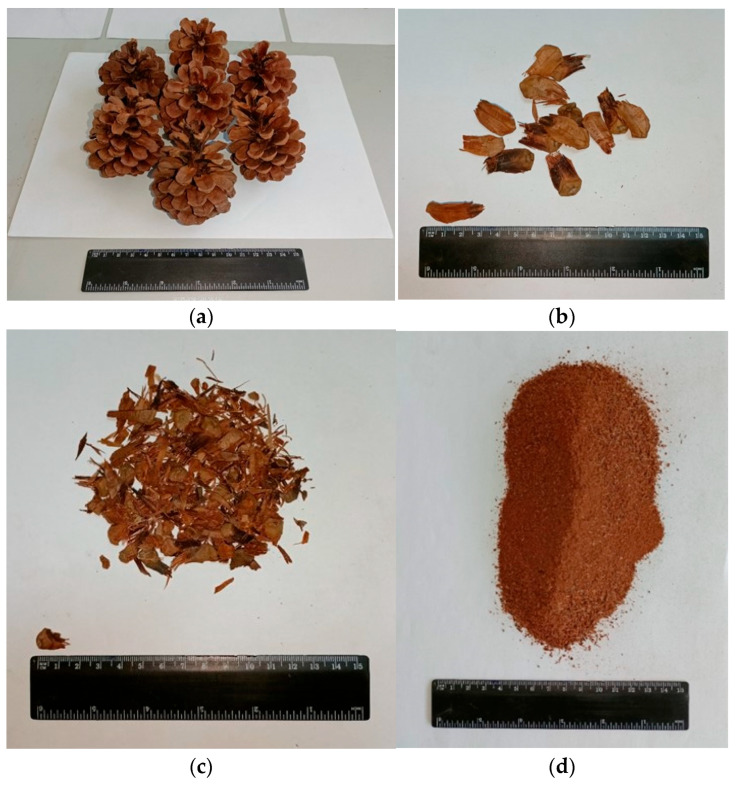
Photos of pine cones, (**a**) After harvesting, (**b**) PCP-4 cm, (**c**) PCP-2 cm, (**d**) PCP-powder.

Pine cones, as shown in Figure 1, were collected at the campus area of Firat University, which is located in Elazig, Turkey. Since the cones were collected in late summer and early autumn, they were dry and already fallen from the tree. After the cones were collected, they were laid out in the laboratory for 14 days to remove the moisture. The dry pine cones were then cut into smaller particles, and then those particles were classified into three groups using a mesh filter.

The first group consisted of pieces that were smashed from the pine cones without being crushed, and their sizes ranged from 2 to 4 cm (designated as PCP-4 cm). The second group was the product of grinding the particles of the first group to reduce their size, so that their sizes ranged between 0 and 2 cm (namely PCP-2 cm). The third group was in the form of powder that was ground to the smallest size (PCP-powder).

### 2.2. Preparation of Pine Tree Resin (PTR)

The PTR is a soft material when on the tree. In time, it becomes sticky and develops a very rigid structure. Dry and pulverized PTR absorbs some water and swells in an aqueous environment. Concrete produced with resin-added cement loses this water as it dries, which results in artificial pores in the microstructure [15].

**Figure 2:**
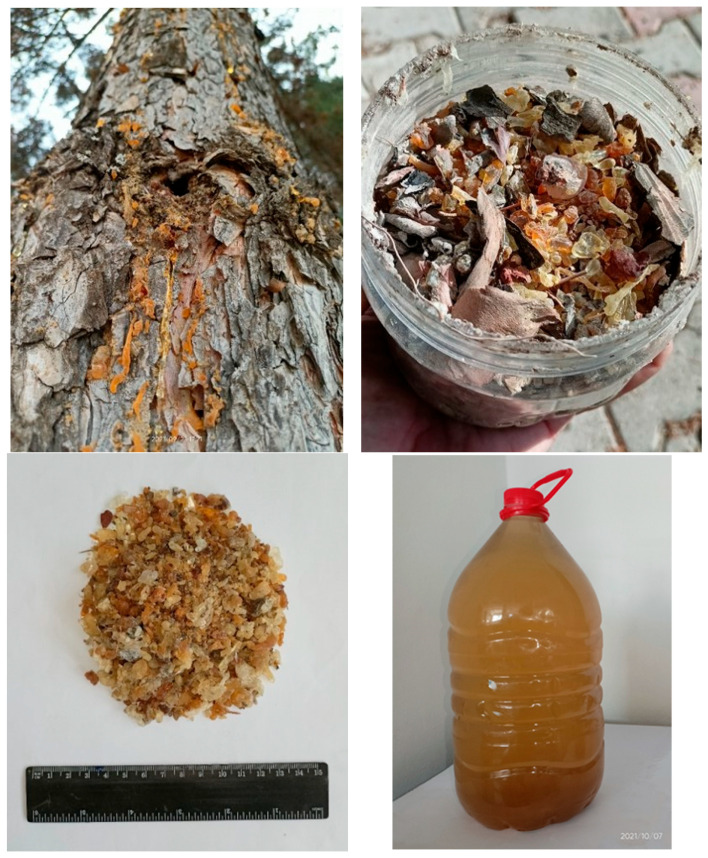
Harvested PTR with impurities, and the final resin solution.

As it is presented in Figure 2, after the impurities were removed, the dry PTR was weighed and then put in a container. In this study, the amount of dry PTR dissolved in the resin solution is 1% of the mass of the final mixture of “cement with PCPs”. The amount of water was determined according to the amount of PTR that would be used in preparing the solution. While working on preparing the resin solution, it was discovered that it is possible to completely dissolve 80 g of dry resin in 5 kg of water. It should be noted that the water content present in the PTR solution was accounted for in the total mixing water calculation. The amount of free water added to the mixture was reduced exactly by the amount of water introduced via the resin solution to ensure the *w/c* ratio remained consistent across all samples.

The resin solution was prepared by (*i*) boiling the resin and (*ii*) washing it with water for several hours and cleaning it from the unwanted substances, (*iii*) crushing, and (*iv*) filtering with a mesh filter. The filtration process removed insoluble particles, sand, and impurity residue from the resin, resulting in a homogeneous product. The consistency of the filtered solution was then such as a resinous or soapy solution diluted with water.

### 2.3. Preparation of the Samples

**Figure 3:**
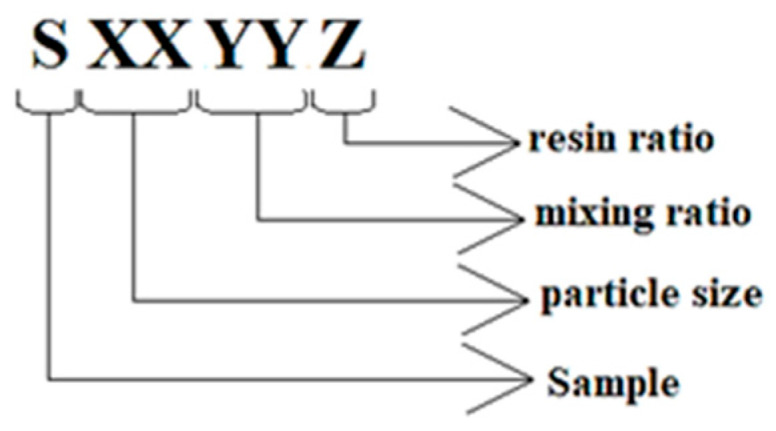
Definition of the samples’ codes.

When preparing the samples, first the PCP-2 cm, PCP-4 cm, and PCP-powder were mixed with cement at a ratio of 20%, 40%, 60%, 80% as shown in Table 1. Then, 1% PTR was added to the PCP + cement mixture. Quantities of materials used in the samples without PTR are listed in Table 2, with PTR are listed in Table 3. Depending on the type of components and mixing proportions, each sample was assigned a code. The codes are explained in Figure 3, and the detailed information is given in Table 4. The codes start with capital S as the Sample, then two digits as XX, referring to the size of the PCP (04, 02, and 00 show the 2–4 cm particles, 0–2 cm particles, and powder form, respectively). YY is the weight ratio of the cement to pine cone (80, 60, 40, and 20). Z refers to the resin ratio in the mixture (1 means the resin ratio is 1%, 0 means there is no resin).

While preparing the samples, first the required amount of PCP was weighed in the measuring cup with a precision balance and put into the mixing bucket. Then the required amount of cement was added to the container. The two materials were mixed with a trowel. Finally, for the samples including PTR, the prepared resin solution was added to the mixture. Casting molds for thermal tests were manufactured (20 × 60 × 150 mm) to match the dimensions of the measuring instrument probe. Similarly, casting molds for mechanical tests were manufactured with a size of 100 × 100 × 100 mm. The water + cement + PCP mixtures with and without PTR solution were poured into the molds. The mortar was put into molds at room temperature, 20 °C, after being well mixed. After keeping them in the mold for 48 h, the samples were removed from the molds and left to dry for 28 days (aging) at room temperature.

**Figure 4:**
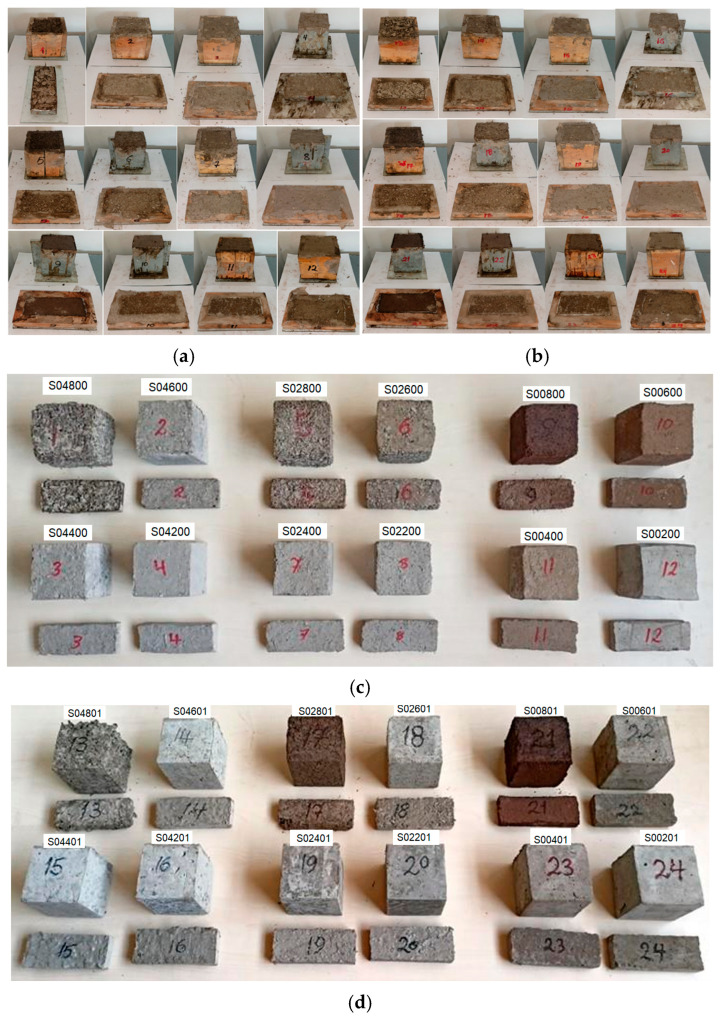
Photos of the samples: (**a**) samples without PTR, when poured into molds, (**b**) Samples with PTR, when poured into molds, (**c**) samples without PTR, after casting, (**d**) samples with PTR, after casting.

Figure 4a,b shows the samples when they are in the molds. Figure 4c,d shows the samples after casting. In Figure 4c,d, the color difference in the samples is due to the proportion and size of the PCP, and the presence of PTR in the mixture. For example, in Figure 4c, the brown sample’s code is S00800, meaning PCP is added to the concrete mixture in powder form at a rate of 80%. Similarly, in Figure 4d, the darkest brown sample’s code is S00801, meaning PCP is added to the concrete mixture in powder form at a rate of 80%, and there is PTR in it. Briefly, the color difference seen in the samples depends on the proportion and shape of the PCP and the presence of PTR.

### 2.4. Methodology

As previously mentioned, all samples were kept in the molds for 28 days, as directed by the American Concrete Institute, “which is the required aging period to apply the compressive strength test on it”. In this way, the samples were dried and prepared to become ready for tests and analysis.

#### 2.4.1. Density Measurements

The density of each sample is the ratio of mass to the volume of the sample.

(1) $$\rho =\frac{m}{V}$$

where *m* is the mass of the dry sample (g), and *V* is the volume of the mold (cm^3^). The mass is measured by the Weather Forecast brand scale, WF Digital precision balance 500 g/0.01 g.

#### 2.4.2. Thermal Measurements

The usability criterion of a building material is its mechanical behavior as well as the improvements it will bring to the building’s heat load. Determination of this is only possible by fully describing the thermal performance of building materials. Parameters defining the thermal performance of a material, such as thermal conductivity, specific heat capacity, and thermal expansion coefficients, can be listed [16].

Accurate and reliable execution of a building’s process simulation or field applications is only possible by accurately measuring the thermal properties of materials. Although there is no standardized method for mostly heterogeneous building materials, there are many methods that measure the thermal properties of homogeneous solid materials using different mathematical and physical principle techniques [17]. The measurement methods with thermal properties can be evaluated in three main classes: steady regime, transient regime, and calorimetric methods [18]. In these methods, measurement samples are generally chosen as plates, cylinders, and spheres [19,20].

Since the structure of the samples, for which the thermal conductivity is being tested, is not homogeneous, and due to the small sample size and short test time, it is easy to test the thermal conductivity without a change in the moisture level. So, the test is made by using the “Isomet 2104” brand test device, which works with a hot wire approach and measures the property in the transient system. This instrument measures thermal conductivity (*k*), specific heat capacity (*C_p_*), and thermal expansion coefficient (α) simultaneously and digitally, using hot-wire methodology according to DIN 51046 [21] standard.

**Figure 5:**
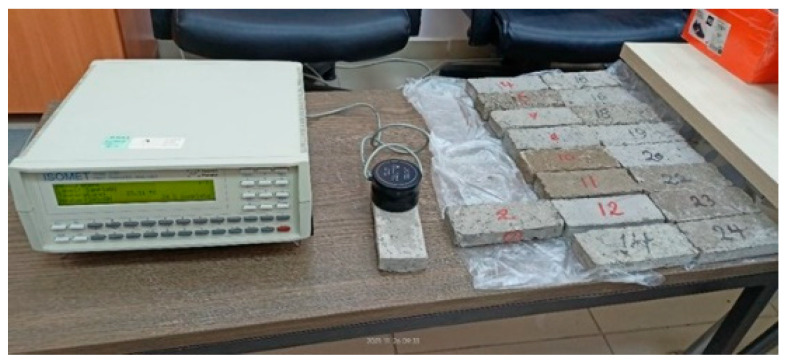
The thermal properties analyzer is used during the testing process.

Test results were taken from 5 different points on each sample with the device in the laboratory at a room temperature of 25 °C. The averages of 3 test values were found to be compatible with each other. With catalog information, the testing apparatus illustrated test results of the thermal conductivity in the range of 0.02–6 W/mK, as shown in Figure 5, with 5% precision, and the volumetric specific heat capacity in the range of 4.0 × 10^4^–4.0 × 10^6^ J/m^3^K with 15% precision.

#### 2.4.3. Mechanical Measurements

**Figure 6:**
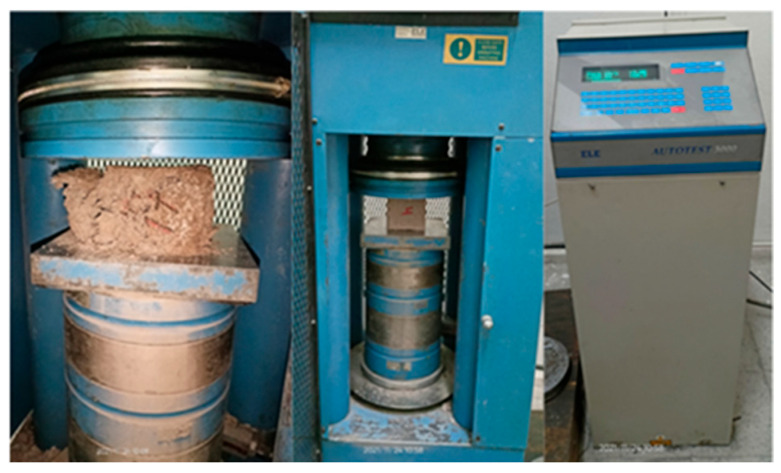
The compressive strength test device.

Compressive strength tests were carried out by using the international brand device (Ele) with a loading capacity of 3000 kN, with a digital control panel and an adjustable loading speed, with the ability to apply force on one axis. Figure 6 shows the test and control units of the device during the testing process.

#### 2.4.4. Water Absorption Measurements

The water absorption tests were carried out to determine the presence of a dry volume in which ice crystals that form inside building materials can expand in direct contact with water at a temperature of 0 °C. This test was carried out in accordance with BS 812 [22] conditions. This test is important, since it ensures that the material will not freeze, increase in size, or cause cracks and failures in these samples after they are used for facade coatings. The dry mass of each sample was determined individually. After that, the samples were placed in a container full of water, so that all the samples in the container were completely immersed.

The samples were taken out of the water and wiped with a cloth to ensure that the water did not stick to the surface of the samples, and then the mass of the samples saturated with water was measured using a sensitive balance. The water absorption rate (*WAR*) for each sample was calculated as follows:

(2) $$WAR=\frac{m_{w}-m_{d}}{m_{d}}\times 100$$

where *m_d_* is the dry mass of the sample (mass of the sample before starting the water absorption test), and *m_w_* is the final mass of each sample, which is kept in the water tank for 24 h.

#### 2.4.5. Drying Rate Measurements

The drying rate of the samples is tested to see if the samples have the property of breathability. The samples were taken out of the water and then wiped with a dry cloth. Then they were left to dry naturally in a room at average room conditions (18 °C and 50% relative humidity) for 48 h. Then the water was removed from the depths of the sample material to the surface through capillary channels, where it dried up by evaporation from the surface. In other words, it dried the body by eliminating moisture by resisting vapor permeability. Drying rate is found as follows:

(3) $$DR=\frac{m_{w}-m_{d}}{m_{w}}\times 100$$

where *m_w_* is the mass of the sample subjected to 24 h of water absorption. *m_d_* is the mass of the sample subjected to 48 h of drying.

**Figure 7:**
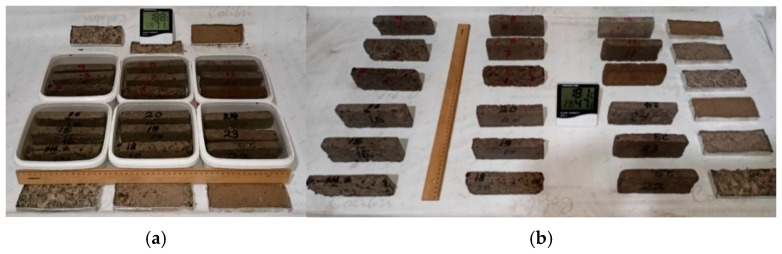
Water tests: (**a**) water absorption (24 h in water), (**b**) drying (48 h in air).

Note that samples with the codes S04800, S02800, S00800, S04801, S02801, and S00801 are not included in the water absorption and drying experiments. This is because they failed the compression test. Figure 7a shows the sample crumbs collected in small aluminum containers. Figure 7b shows the samples’ photos after leaving them to dry for 48 h. It should provide a concise and precise description of the experimental results, their interpretation, and the experimental conclusions that can be drawn.

## 3. Results

In this study, initially, 12 concrete samples were produced by adding PCP water and cement, namely without PTR. Then, 12 samples were produced by adding 1% of PTR to the mixture of PCP water and cement. All 24 samples were applied to the aforementioned tests in the previous section. It should be noted that all samples containing 80% PCP were discarded because they had failed after hardening and drying, and had a predetermined lifespan that exceeded 28 days. The reason was its fragility, poor skeletal structure, and lack of consistency in a single solid mass.

### 3.1. Results of Thermal Conductivities

**Figure 8:**
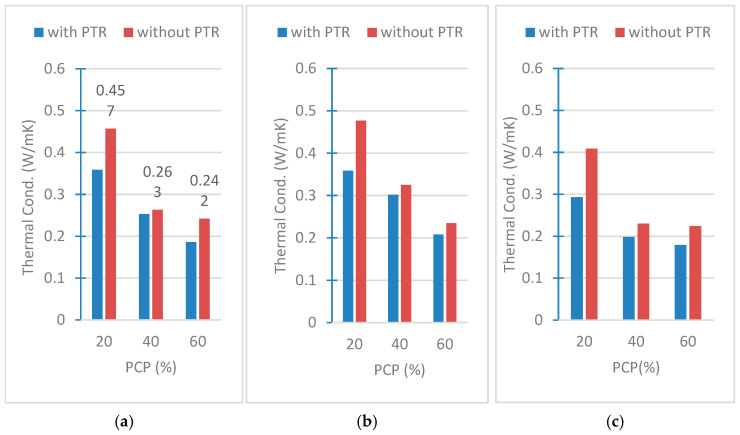
Thermal conductivities of (**a**) PCP-4 cm, (**b**) PCP-2 cm, (**c**) PCP-powder.

Thermal conductivity has received significant attention among the various thermal properties of concrete, as it depends on the composition of the material and plays a crucial role in building insulation by measuring the ability of a material to transfer heat [23]. Figure 8a–c presents the variations in the thermal conductivities, respectively, for PCP-4 cm, PCP-2 cm, and PCP-powder.

When we analyze Figure 8a–c, we see that the highest thermal conductivity of all samples is measured as 0.477 W/mK for the sample S02200. It means that the highest thermal conductivity was measured in resin-free samples, with 20% of the participation rate of PCP-2 cm. The smallest thermal conductivity is measured as 0.179 W/mK for the sample S00601, which contains 1% PTR and 60% PCP-powder in its composition, and in which the result was lower than the result of the same sample without resin.

These results show that the effect of PCP and PTR on the thermal conductivity of the material is significant. The presence of PTR decreased the thermal conductivity values at all PCP ratios. Thermal conductivity generally decreases with the increase in the PCP ratio, but this decrease differs according to the presence or absence of PTR. While PCP-4 cm, the effect of PTR is more pronounced, especially at low PCP ratios (20%), and this difference decreases as the PCP ratio increases. The effect of PTR is felt more clearly while PCP-2 cm. Especially at 20% and 40% PCP ratios, a significant difference occurs between the cases with and without PTR. In the PCP powder, it was observed that the effect of PTR decreased to a minimum level at 40% PCP.

These results reveal that PTR is a critical parameter affecting the thermal conductivity and that the ratio and form of PCP significantly shape this effect. As a result, the thermal insulation performance can be optimized by considering the presence of PTR and the size of PCP together. It should also be added that the decrease in conductivity because of adding PCP into the mixture can be explained by the shrinkage caused by the loss of water during mold casting and the 28-day drying process. The lost water led to the formation of pores according to the amount of PCP.

Compared to conventional aggregates, samples with PCP show lower and acceptable thermal conductivity. However, as all tested samples exceed the ISO threshold of 0.065 W/mK [24] for heat-insulating materials, they are classified as lightweight concrete, particularly those with 20%, 40%, and 60% pine granules (0.060–0.610 W/mK).

### 3.2. Results of Specific Heat Capacities

**Figure 9:**
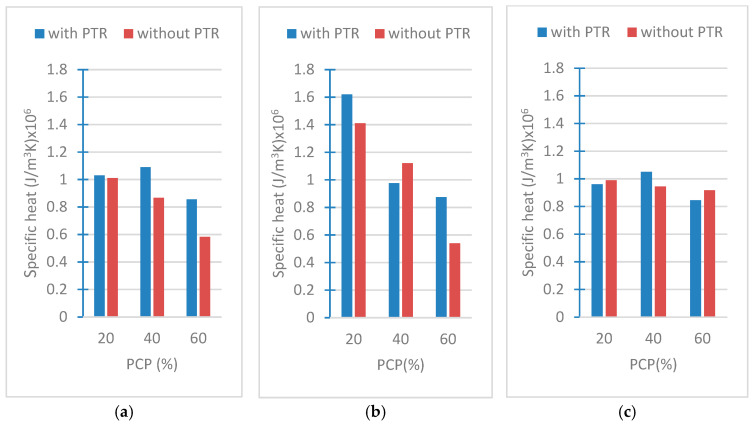
Specific heat capacities of (**a**) PCP-4 cm, (**b**) PCP-2 cm, (**c**) PCP-powder.

Specific heat capacity is an important property for thermal comfort calculations and building insulation classification, as it influences the heat storage capacity of building components. To enhance thermal insulation, the specific heat capacity of materials should be minimized. The specific heat capacity shows the frequency of completion of the heating or cooling process. The results of the heat capacity tests are shown in Figure 9a–c. It should be noted that as the weight ratio of PCP increases, the specific heat decreases. Additionally, the presence of PTR in the samples reduces the specific heat for the majority of samples.

The results show that the presence of PTR reduces the specific heat capacity of the material and thus has the potential to improve the energy storage capacity. Especially at low PCP ratios (20%), the effect of PTR was observed quite clearly. In this case, it can be said that PTR enhances the thermal efficiency of the concrete by increasing the specific heat capacity. As the PCP ratio increases (40% and 60%), the effect of PTR on the specific heat capacity gradually decreases. The presence of PTR still creates a significant difference at 40% PCP ratio, but this difference decreases to a minimum level at 60% PCP ratio. This trend suggests that high PCP ratios may limit the effectiveness of PTR on the material. It can be inferred that PCP at high ratios creates a saturation effect in the internal structure of the material, limiting the thermal improvement capacity that PTR can exhibit.

The form of PCP also stands out as an important factor affecting the specific heat capacity. Larger particles (e.g., PCP-4 cm and PCP-2 cm) increase the effect of PTR, while it is observed that the effect of PTR decreases significantly in PCP powder form. Larger particles may allow PTR to interact more effectively with the material. In powder form, it is seen that the effect of PTR in the material is dispersed with the increase in surface area, and, therefore, the potential to increase specific heat capacity decreases.

These results reveal that the combined use of PCP and PTR should be carefully designed to optimize the thermal performance of the material. Low PCP ratios and larger particle sizes may provide an ideal structure to maximize the effect of PTR. On the other hand, the use of high ratios of PTR with PCP in powder form creates a situation where these effects are limited. These findings are important in terms of sustainable material design and show that PCP and PTR can be used in the development of energy-efficient and environmentally friendly materials.

In the summer, because of the high temperatures, the high heat from the sun on the outer walls is stored in this wall material as a thermal mass that is returned to the inside and outside at night. In winter, the external ambient temperature is significantly lower than the internal temperature. Due to this large thermal gradient, a significant amount of heat leaks out through the same mechanism observed in summer, but in the opposite direction [10]. The high heat capacity of the outer wall causes unnecessary energy consumption. For this reason, a decrease in the heat capacity of the samples with an increase in the proportion of PCPs in their composition would be a benign and desirable characteristic.

### 3.3. Results of Thermal Expansion Coefficients

**Figure 10:**
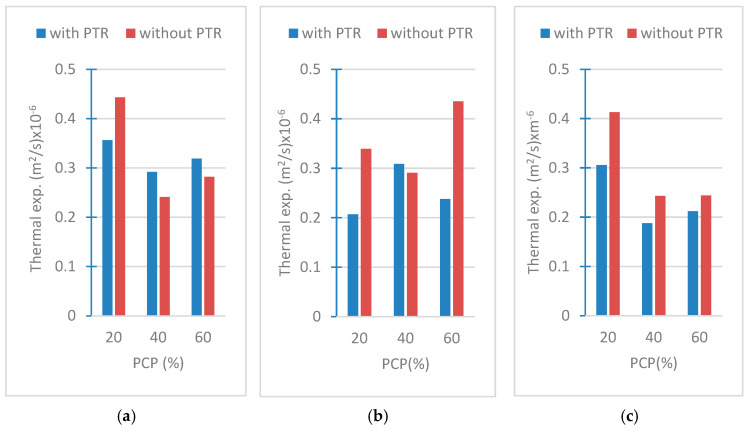
Thermal expansion coefficients of (**a**) PCP-4 cm, (**b**) PCP-2 cm, (**c**) PCP-powder.

The thermal expansion coefficient of concrete is important for understanding how concrete will behave under temperature fluctuations, especially in large structures such as bridges, roads, or buildings, where thermal expansion can induce significant stresses. Engineers use this value to ensure that joints and connections can accommodate thermal movement without causing cracking or other damage. In the present study, the thermal expansion coefficients are displayed in Figure 10.

The thermal expansion coefficient is a parameter that expresses how a material responds dimensionally to temperature changes. The magnitude of this coefficient indicates the material’s tendency to change dimensions in response to temperature changes. High thermal expansion coefficient values indicate that the material expands more when the temperature increases or contracts more when it decreases. Low values indicate that the material is more stable to temperature changes. The presence of PTR generally reduces the thermal expansion, making the material more stable to temperature changes. This shows that PTR has the potential to increase structural durability by regulating the expansion behavior of the material. It is seen that the coefficient of thermal expansion generally decreases as the PCP ratio increases. At lower PCP ratios (20%), the coefficient of thermal expansion is higher, indicating that the material is more sensitive to temperature changes. However, when the PCP ratio increases up to 60%, it is observed that the coefficient of expansion decreases, indicating that the material becomes more stable against temperature changes.

The effect of PTR varies depending on the form of PCP. While the effect of PTR in reducing the thermal expansion coefficient is more pronounced at larger particle sizes (PCP-4 cm), it is observed that this effect decreases in the powder form of PCP. This can be explained by the fact that PCP in powder form increases the interaction surface with PTR and thus disperses the stability-increasing effect of PTR. As a result, a low coefficient indicates that the material is more resistant and stable against temperature changes. In this study, the presence of PTR significantly reduced the thermal expansion coefficient, and the magnitude of this effect varied according to the PCP ratio and size.

### 3.4. Results of Water Absorption Tests

**Figure 11:**
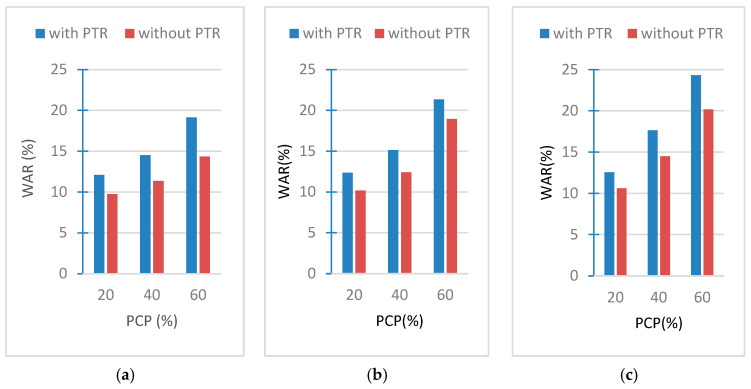
Water absorption ratios of (**a**) PCP-4 cm, (**b**) PCP-2 cm, and (**c**) PCP-powder.

The water absorption rates of the samples are found throughout a 24 h water immersion period. The data gained from the samples’ water absorption tests are plotted in Figure 11a–c. After completing the examination of the water absorption tests, it has been discovered that the water absorption percentage of the samples is less than 24.31% [25]. Therefore, it is clear that all concrete samples can be used for internal partition walls or external facades when protected by appropriate plaster or coating, and there will be no danger of freezing, cracking, or scaling at temperatures below 0 °C.

**Figure 12:**
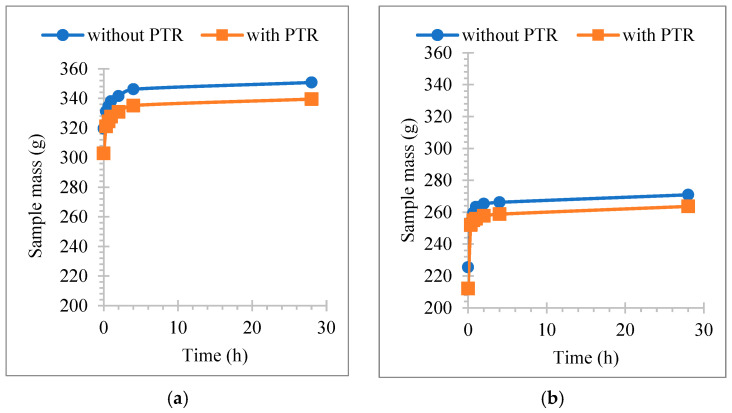
Variation in water absorption with time (**a**) PCP-4 cm 20%, (**b**) PCP-powder, 60%.

Figure 12a,b show the variation in water absorption ratio with time. It would be useful to point out here that we have graphically presented the two cases where the highest and lowest values were obtained among all cases. It is clear from both figures that the rate of water absorption of the samples is at its peak in the first hour from the start of the test and decreases gradually after that. The samples absorb water through capillary channels in their internal structure. Because the sample is completely dry, the absorption process at its beginning is as fast as soon as its surfaces come into contact with water, so its mass increases. Then the process begins to slow down in the rate of water absorption because the sample reaches saturation with water.

The water absorption rates depend on the variables of this research. The first influencing factor is the weight ratio of PCPs, as the water absorption rates in the samples increase with this ratio. This is because pine cones are fibrous, porous, and become wet (absorb water) once exposed to moisture. The second factor that affects the absorption is the PCP size; it was found that the smaller the size of these particles, the greater the percentage of absorption, so the relationship between them is inverse. The third factor that was found to affect the water absorption is PTR, as it was found that PTR leads to an increase in the rate of water absorption in the same sample that is free of resin.

### 3.5. Results of Drying Rate Tests

**Figure 13:**
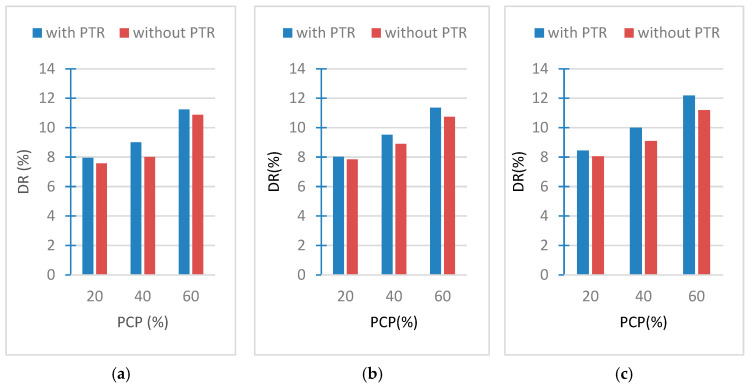
Drying rate of (**a**) PCP-4 cm, (**b**) PCP-2 cm, (**c**) PCP-powder.

The results of the experiments carried out to check the breathing capacity of the samples are shown in Figure 13a–c. The results showed that the drying rate of samples within 48 h changes with the weight ratio of PCPs. It is seen that the highest drying rate in the samples was 12.18%, and PCP-powder had the highest participation for the proportion of the amount of pine cone powder (60%) in the composition of the sample, while it contained PTR in its composition. While the lowest drying rate of the samples from the absorbed water was 7.57%, and this percentage was found in the sample that contains the properties of PCP-4 cm, the lowest weight ratio 20%, and without PTR.

**Figure 14:**
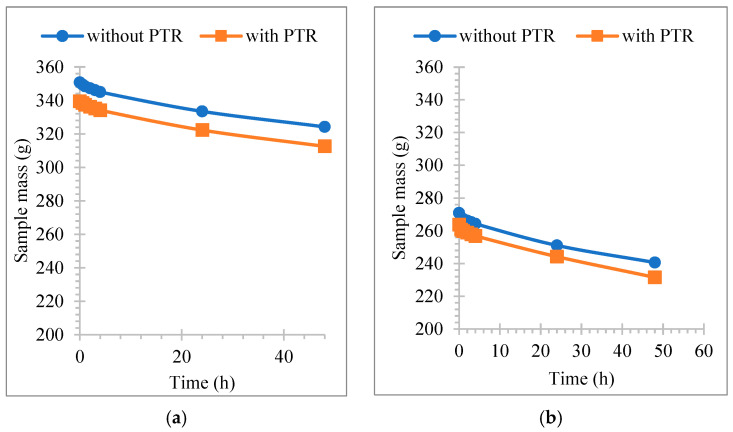
Variation in drying time (**a**) PCP-4 cm 20%, (**b**) PCP-powder, 60%.

Figure 14a,b show the mass change in samples over a 24 h period. The water loss towards the surfaces of the samples is through the pores and capillary channels formed inside them; it occurs faster in the first four hours of the drying test process, as shown in the diagram, where the drying curve is steeper than the rest of the hours, and then occurs gradually until completely dry, the rest of the time the drying process of the samples is at a slower pace.

When the weight ratio of PCPs is high, drying occurs faster. Additionally, when the PCP sizes are smaller, the drying rate in the sample is higher. The presence of PTR leads to an increase in the drying of the absorbed moisture compared to the same sample free of PTR, due to the pores and capillary channels formed by the PTR.

### 3.6. Results of Mechanical Tests

**Figure 15:**
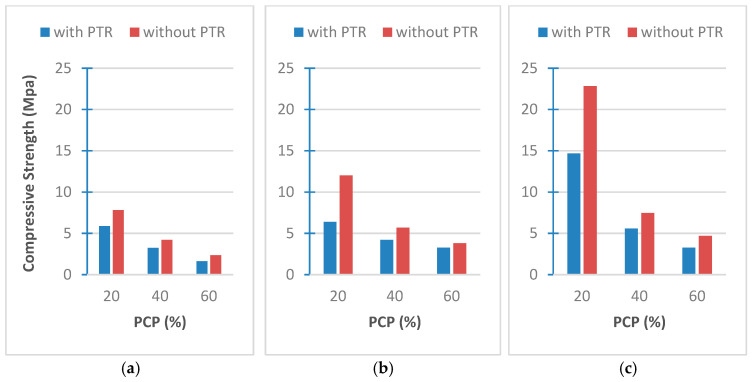
Compressive strength of (**a**) PCP-4 cm, (**b**) PCP-2 cm, (**c**) PCP-powder.

Figure 15a–c present the compressive strength tests. It is clear that samples with a large size of PCP and the presence of PTR lead to a decrease in compressive strength. In other words, the presence of PTR, the ratio of PCP, and the size of PCP have a similar effect. The larger they are, the lower the compressive strength. This can be attributed to two reasons: first, the shrinkage of the proportion of the binder material (cement) with the increase in the proportion of PCPs, and the second reason is due to the artificial pores formed by the resin in the area of the binder, which leads to its weakness.

The results can also be compared to similar studies. Subasi [25] measured the compressive strength as 16.67, 23.27, and 41.27 MPa for samples of expanded clay and cement, whose densities were 1.370, 1.560, and 1.700 g/cm^3^, respectively. Benazzouk et al. [26] measured compressive strengths as 23.30, 16.00, and 10.50 MPa for samples with rubber bits and cement with densities of 0.625, 0.516, and 0.470 g/cm^3^, respectively. Khedari et al. [27] measured compressive strengths as 1.97 and 2.53 MPa by mixing cement and coconut fiber with densities of 0.770 and 1.106 g/cm^3^, respectively. Al Rim et al. [28] tested compressive strength as 2.67, 2.35, and 1.35 MPa for samples of cement and wood chips with densities of 1.010, 0.870, and 0.700 g/cm^3^, respectively. These comparisons show that, although our results are not high enough, they are similar to the newly produced bio-composite concretes in the literature.

### 3.7. Results of Density Tests

**Figure 16:**
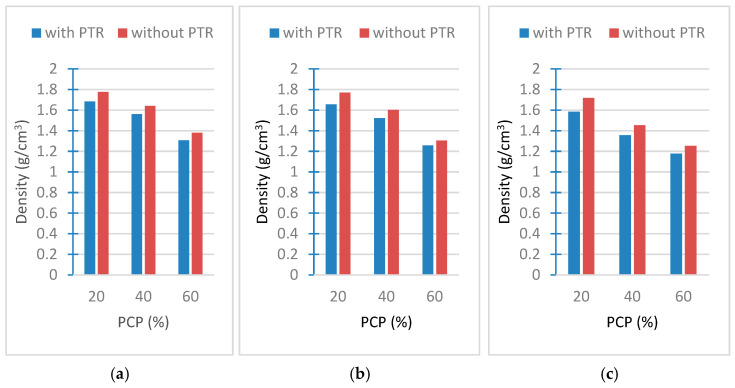
Density versus weight ratio of PCP (**a**) PCP-4 cm, (**b**) PCP-2 cm, and (**c**) PCP-powder.

Figure 16a–c show the result of density tests. It is seen that the density values decrease as the PCP increases. The reason for this is that the resin added to the sample in solution causes artificial pores during the drying process. Therefore, the density becomes smaller. The measured densities of PCP-4 cm, PCP-2 cm, and PCP-powder were found to be 0.163 g/cm^3^, 0.185 g/cm^3^, and 0.322 g/cm^3^, respectively.

## 4. Conclusions

The results of compressive strength tests show that concretes with PCP are unsuitable for load-bearing elements such as columns and beams. However, they can still function as carrier concrete if PCPs are partially replaced with natural aggregates or strengthened with additives such as waste plastic or steel fibers. This study aims to investigate the usage area of pine cones and pine tree resin in the production of lightweight concrete materials.

Experimental results indicate that thermal conductivity, specific heat capacity, and the thermal expansion coefficient decrease as the PCP ratio and particle size increase. The incorporation of PTR increases porosity, which further reduces these thermal properties for the majority of samples. The mechanism behind these changes is attributed to the hydrophobic nature of the pine resin bio-polymer. The resin likely forms a film around the cement particles or entraps air during mixing, which modifies the hydration process. This interaction results in the observed pore formation, reducing thermal conductivity while maintaining the material’s breathability. Although compressive strength values decrease with the addition of PTR and PCP, water absorption rates remain below the critical 30% threshold. This suggests the material is suitable for applications such as infill walls or insulated blocks protected from direct weather exposure. Additionally, drying rates are initially rapid in the first two hours before stabilizing linearly.

Evaluating these findings, it can be concluded that pine cones are a surprisingly useful material for lightweight construction. Their unique structure, which is both lightweight and relatively strong, effectively reduces the overall weight of building materials while maintaining sufficient strength. Furthermore, the cellular structure inside the cones traps air, providing excellent thermal and acoustic insulation properties, making them suitable for incorporation into wall panels or as fillers in insulation products. When broken down and dried, pine cones can potentially serve as aggregates in lightweight concrete or mortar. Their air-filled cellular structure allows them to act as lightweight fillers that reduce concrete density while preserving structural integrity. Consequently, crushed pine cones could be incorporated into foam concrete for insulation and lightweight applications, resulting in lighter and more eco-friendly materials.

Based on this study, it is recommended to further explore the use of pine tree resin with pine cones to form bio-composites. These composites show potential for use in construction elements such as panels, insulation, or structural components, offering a sustainable and lightweight alternative to traditional concrete or wood. Additionally, pine cones can be compressed into pellets to create lightweight boards or panels. These materials could be employed in partition walls, flooring, or decorative elements, providing a renewable alternative to synthetic materials.

## Figures and Tables

**Table 1:** Mass (g) of the samples.

Materials	Mass of the Materials in the Mixture (g)			
	20%	40%	60%	80%
PCP-4 cm	95	190	285	380
PCP-2 cm	125	250	375	500
PCP-powder	190	380	570	760
Cement	620	1240	1860	2480

**Table 2:** Quantities of materials used in the samples without PTR.

PCP (%)	Cement (%)	PCP (g)	Cement (g)	Total Mix. (g)
80	20	380	620	1000
60	40	285	1240	1525
40	60	190	1860	2050
20	80	95	2480	2575
80	20	500	620	1120
60	40	375	1240	1615
40	60	250	1860	2110
20	80	125	2480	2605
80	20	760	620	1380
60	40	570	1240	1810
40	60	380	1860	2240
20	80	190	2480	2670

**Table 3:** Quantities of materials used in the samples with PTR (1%).

Weight Ratios (%)			Mass in (g)			
PCP (%)	Cement (%)	PTR (%)	PCP (g)	Cement (g)	Total Mix. Mass (g)	PTR (g)
80	20	1	380	620	1000	10
60	40	1	285	1240	1525	15.25
40	60	1	190	1860	2050	20.5
20	80	1	95	2480	2575	25.75
80	20	1	500	620	1120	11.2
60	40	1	375	1240	1615	16.15
40	60	1	250	1860	2110	21.1
20	80	1	125	2480	2605	26.05
80	20	1	760	620	1380	13.8
60	40	1	570	1240	1810	18.1
40	60	1	380	1860	2240	22.4
20	80	1	190	2480	2670	26.7

**Table 4:** Codes of the samples according to their ingredients.

**PCP-4 cm**			
**Code**	**PTR (%)**	**PCP (%)**	**Cement (%)**
S04800	0	80	20
S04600		60	40
S04400		40	60
S04200		20	80
S04801	1	80	20
S04601		60	40
S04401		40	60
S04201		20	80
**PCP-2 cm**			
**Code**	**PTR (%)**	**PCP (%)**	**Cement (%)**
S02800	0	80	20
S02600		60	40
S02400		40	60
S02200		20	80
S02801	1	80	20
S02601		60	40
S02401		40	60
S02201		20	80
**PCP-Powder**			
**Code**	**PTR (%)**	**Powder (%)**	**Cement (%)**
S00800	0	80	20
S00600		60	40
S00400		40	60
S00200		20	80
S00801	1	80	20
S00602		60	40
S00403		40	60
S00204		20	80

## Data Availability

The data presented in this study is available on request from the corresponding author. The original contributions presented in this study are included in the article. Further inquiries can be directed to the corresponding author.

## References

[1] Cetiner I., Shea A.D. (2018). Wood waste as an alternative thermal insulation for buildings. Energy Build..

[2] Korjenic A., Petránek V., Zach J., Peterkova J. (2011). Development and performance evaluation of natural thermal-insulation materials composed of renewable resources. Energy Build..

[3] Bayraktar O.Y., Ozel H.B., Benli A., Yılmazoglu M.U., Turkel I., Dal B.B., Sevik H., Kaplan G. (2024). Sustainable foam concrete development: Enhancing durability and performance through pine cone powder and fly ash incorporation in alkali-activated geopolymers. Constr. Build. Mater..

[4] Singh A., Yadav B.P., Giri B.S. (2025). Pine cone waste from pine tree as a sustainable coarse aggregate for lightweight concrete: Physical and mechanical properties. Proc. Indian Natl. Sci. Acad..

[5] Arrakhiz F.Z., El Achaby M., Benmoussa K., Bouhfid R., Essassi E.M., Qaiss A. (2012). Evaluation of mechanical and thermal properties of Pine cone fibers reinforced compatibilized polypropylene. Mater. Des..

[6] Agayev S., Ozdemir O. (2019). Fabrication of high density polyethylene composites reinforced with pine cone powder: Mechanical and low velocity impact performances. Mater. Res. Express.

[7] Efe F.T. (2022). Investigation of some physical and thermal insulation properties of honeycomb-designed panels produced from *Calabrian pine* bark and cones. Eur. J. Wood Wood Prod..

[8] Basturk B., Kanbur K., Polatoglu I., Yurekli Y. (2015). Mechanical properties of acorn and pine cone filled polymer composites. Am. Sci. Res. J. Eng. Technol. Sci..

[9] Ayrilmis N., Buyuksari U., Avci E., Koc E. (2009). Utilization of pine (*Pinus pinea* L.) cone in manufacture of wood based composite. For. Ecol. Manag..

[10] Bicer A., Kar F. (2017). The effects of apricot resin addition to the light weight concrete with expanded polystyrene. J. Adhes. Sci. Technol..

[11] McSwiggan C., Mak K., Fam A.M. (2017). Concrete bond durability of CFRP sheets with bioresins derived from renewable resources. J. Compos. Constr..

[12] Ayse K., Filiz K. (2016). Properties of concrete containing waste expanded polystyrene and natural resin. Constr. Build. Mater..

[13] Bicer A. (2021). The effect of fly ash and pine tree resin on thermo-mechanical properties of concretes with expanded clay aggregates. Case Stud. Constr. Mater..

[14] Devecioglu A.G., Bicer Y. (2016). The effects of tragacanth addition on the thermal and mechanical properties of lightweight concretes mixed with expanded clay. Period. Polytech. Civ. Eng..

[15] Babu K.G., Babu D.S. (2003). Behaviour of lightweight expanded polystyrene concrete containing silica fume. Cem. Concr. Res..

[16] Bouguerra A., Aït-Mokhtar A., Amiri O., Diop M.B. (2001). Measurement of thermal conductivity, thermal diffusivity and heat capacity of highly porous building materials using transient plane source technique. Int. Commun. Heat Mass Transf..

[17] Kočí V., Maděra J., Jerman M., Trník A., Černý R. (2014). Determination of the equivalent thermal conductivity of complex material systems with large-scale heterogeneities. Int. J. Therm. Sci..

[18] Czichos H., Saito T., Smith L. (2011). Springer Handbook of Metrology and Testing.

[19] Andrianov I., Danishevskyi V., Tokarzewski S. (1996). Two-point quasifractional approximants for effective conductivity of a simple cubic lattice of spheres. Int. J. Heat Mass Transf..

[20] Rides M., Morikawa J., Halldahl L., Hay B., Lobo H., Dawson A., Allen C. (2009). Intercomparison of thermal conductivity and thermal diffusivity methods for plastics. Polym. Test..

[21] https://www.dinmedia.de/en/pre-standard/din-51046-1/596015.

[22] http://www.saliergeotechnical.co.uk/Aggregates/BS_BDVAB_812/BS%20812%20Part%202%201995.pdf.

[23] Misri Z., Ibrahim M.H.W., Awal A.S.M.A., Desa M.S.M., Ghadzali N.S. (2018). Review on factors influencing thermal conductivity of concrete incorporating various type of waste materials. IOP Conf. Ser. Earth Environ. Sci..

[24] https://cdn.standards.iteh.ai/samples/70322/81157e83dd9544bbbc00586b6e7710c2/ISO-4898-2018.pdf.

[25] Subasi S. (2009). Production of structural lightweight concrete with expanded clay aggregate. J. Eng. Archit. Fac. Gazi Univ..

[26] Benazzouk A., Douzane O., Mezreb K., Laidoudi B., Quéneudec M. (2008). Thermal conductivity of cement composites containing rubber waste particles: Experimental study and modelling. Constr. Build. Mater..

[27] Khedari J., Suttisonk B., Pratinthong N., Hirunlabh J. (2001). New lightweight composite construction materials with low thermal conductivity. Cem. Concr. Compos..

[28] Al Rim K., Ledhem A., Douzane O., Dheilly R., Queneudec M. (1999). Influence of the proportion of wood on the thermal and mechanical performances of clay-cement-wood composites. Cem. Concr. Compos..

